# Surgical vs Transcatheter Aortic Valve Replacement for Asymptomatic Severe Aortic Stenosis- an Updated Meta-Analysis of Reconstructed Individual Patient Data

**DOI:** 10.1093/icvts/ivaf308

**Published:** 2025-12-24

**Authors:** Ahmed K Awad, Ahmed Samy Badran, Ahmed R Gonnah, Ahmed Essam Helmy, Ayman K Awad, Mohammed A Elbahloul, Abdullah K Alassiri, Asad Saulat Fatimi, Sriharsha Talapaneni, Meghna Khandelwal, Irbaz Hameed

**Affiliations:** Department of Cardiothoracic Surgery, Ain Shams University Hospital, Cairo, 11566, Egypt; Faculty of Medicine, Ain-Shams University, Cairo, 11566, Egypt; Department of Cardiothoracic Surgery, Ain Shams University Hospital, Cairo, 11566, Egypt; Faculty of Medicine, Ain-Shams University, Cairo, 11566, Egypt; Department of Medicine, Imperial College Healthcare NHS Trust, London, SW7 2AZ, United Kingdom; Faculty of Medicine, Kafr El-Shaikh University, Kafr El Shaikh, 33511, Egypt; Department of Cardiothoracic Surgery, Ain Shams University Hospital, Cairo, 11566, Egypt; Faculty of Medicine, Ain-Shams University, Cairo, 11566, Egypt; Faculty of Medicine, Kafr El-Shaikh University, Kafr El Shaikh, 33511, Egypt; Faculty of Medicine, King Abdulaziz University, Jeddah, 21589, Saudi Arabia; Department of Cardiothoracic Surgery, Yale University New Haven Medical Center, Connecticut, 06519, United States; Department of Cardiothoracic Surgery, Yale University New Haven Medical Center, Connecticut, 06519, United States; Department of Cardiothoracic Surgery, Yale University New Haven Medical Center, Connecticut, 06519, United States; Department of Cardiothoracic Surgery, Yale University New Haven Medical Center, Connecticut, 06519, United States

**Keywords:** severe aortic stenosis, valve replacement, conservative management

## Abstract

**Objectives:**

The management of asymptomatic aortic stenosis (AS) is controversial. We perform a meta-analysis of reconstructed individual patient data to compare conservative treatment versus transcatheter or surgical aortic valve replacement (TAVR or SAVR) in asymptomatic AS.

**Methods:**

PubMed/MEDLINE, Web of Science, Scopus, and Cochrane CENTRAL were systematically searched, through 25^th^ September 2025, to identify any observational or randomized controlled studies that comply with our inclusion criteria. Our primary outcomes were short cardiovascular and non-cardiovascular mortality as well as long-term mortality. We combined aggregate data meta-analysis for dichotomous outcomes using incidence rate ratio (IRR) with reconstructed time-to-event data derived from published Kaplan–Meier curves using validated algorithms.

**Results:**

Seventeen studies were included. Overall, AVR was associated with a significantly lower risk of all-cause mortality compared with conservative management (IRR = 0.43; 95% CI, 0.32-0.57; *P* < .001). Cardiovascular mortality was also significantly reduced with AVR (IRR = 0.47; 95% CI, 0.29-0.75; *P* < .01; I^2^ = 62%). The risk of heart failure hospitalization was markedly lower among AVR recipients (IRR = 0.39; 95% CI, 0.22-0.67; *P* < .01), with consistent benefit across study designs. AVR was further associated with a substantial reduction in sudden cardiac death (IRR = 0.13; 95% CI, 0.04-0.48; *P* < .01) and myocardial infarction (IRR = 0.11; 95% CI, 0.04-0.31; *P* = .03). The risk of stroke was not significantly different between groups (IRR = 0.77; 95% CI, 0.58-1.02; *P* = .07). Reconstructed Kaplan–Meier analyses demonstrated durable long-term survival benefit with AVR, consistent across surgical and transcatheter approaches.

**Conclusions:**

Patients with asymptomatic AS, managed with either SAVR or TAVR, were associated with lower short- and long-term mortality compared to conservative management.

## Graphical abstract

**Figure:**
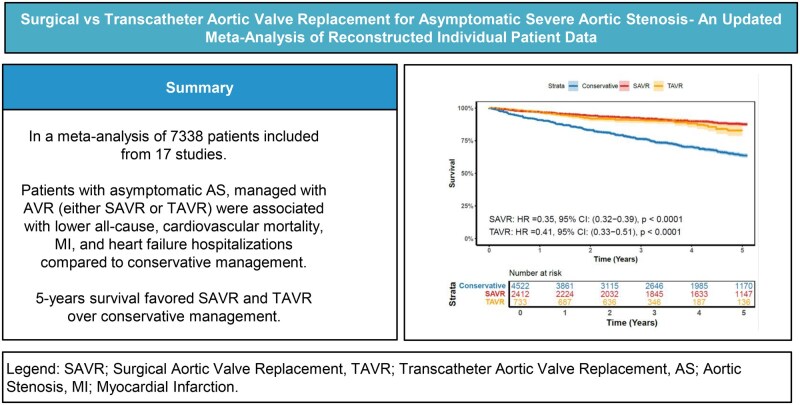

## Introduction

The American Heart Association/American College of Cardiology (AHA/ACC)^1^ and European Society of Cardiology/European Association of Cardiothoracic Surgery (ESC/EACTS)^2^ Valvular heart guidelines recommend aortic valve replacement (AVR) in asymptomatic patients only in the presence of associated heart left ventricular dysfunction, abnormal stress test result, very severe aortic stenosis (AS) (defined as Vmax greater or equal 5.0 m/s or mean transaortic gradient 60 mmHg), and rapid progression of AS (defined as an increase in Vmax 0.3 m/s per year). Furthermore, ESC/EACTS guidelines state that raised b-natriuretic peptide levels (3 SD above the age/sex limit) and severe pulmonary hypertension (defined as pulmonary artery systolic pressure 60 mmHg at rest) are rational indications for AVR.^2^ Asymptomatic AS, however, is often managed conservatively with medical therapy because of the relatively innocuous course, ie, the risk of sudden death is low (<1% per year). The severity of AS can progress rapidly, leading to irreversible negative cardiac remodelling.

The appropriate management for asymptomatic severe AS through surgical and/or transcatheter aortic valve replacement (SAVR and TAVR) remains controversial. Recent analyses support the potential role of early AVR as a better choice than watchful waiting^3–5^ yet without shedding the light on long term outcomes especially mortality.

We conducted our meta-analysis to compare early surgical or transcatheter valve replacement with conservative management in severe asymptomatic AS patients. By investigating the currently available evidence-including the recently published EVOLVED and EARLY TAVR^6^^,^^7^ trials, we aimed to assess the impact of each approach on outcomes both on short and long term with as well a reconstructed individual patient data (IPD) to enhance prognostic insights.

## Materials and methods

This review was performed according to the Preferred Reporting Items for Systematic Reviews and Meta-Analysis (PRISMA) guidelines.^8^ Furthermore, our study was registered in Open Science Framework (OSF) with DOI: 10.37201/OSF.IO/TQCXA.

### Search strategy and screening

Systematic searches were performed from inception to September 25, 2025, on the following databases: PubMed/MEDLINE, Web of Science, Scopus, and Cochrane CENTRAL. The search strategy included broad terms for: aortic stenosis (AS) and conservative treatment. The complete strategy for each database is available in the (**Table S1**). Manual searches in reference lists of included studies were also performed. Titles and abstracts of retrieved articles were screened for eligibility after duplicate removal. Relevant articles were evaluated for full-text screening, and those fulfilling our eligibility criteria had their data extracted. The screening process was done by two independent authors (A.K.A, M.A.E), and a third author (A.E.H) assisted with judgement and resolving conflicts.

### Eligibility criteria and endpoints

Our eligibility criteria are (1) Studies whether observational studies or randomized controlled trials (RCTs) that assessed the efficacy of early valve replacement either surgical or transcatheter in severe asymptomatic AS versus conservative treatment, (2) published in international peer-reviewed journals, (3) no restriction to a certain population, (4) English language papers. Moreover, our PICO format was P: patients with asymptomatic severe aortic stenosis, I: early intervention, either surgical or transcatheter, C: conservative management, O: primary outcomes were all-cause, cardiovascular, and non-cardiovascular mortality, while secondary outcomes were Myocardial infarction (MI), stroke, and sudden death. We excluded any other studies that did not match our criteria. We excluded animal studies, reviews, case reports, abstracts, letters to the editor, and unretrieved full-text articles. When multiple publications reported data from overlapping populations, only the most recent and complete dataset was retained.

### Data extraction

Two reviewers (U.A. and M.A.E.) independently extracted data using a standardized form. Extracted information included study-level characteristics (first author, year of publication, design, country, setting, sample size, study duration, inclusion criteria, primary outcomes, and intervention approach) and patient-level baseline characteristics (number of participants, follow-up duration, mean age, sex distribution, body mass index [BMI], and cardiovascular comorbidities including hypertension, diabetes mellitus, coronary artery disease, atrial fibrillation, and chronic lung disease). Discrepancies were resolved through discussion or adjudication by a third investigator (A.K.A.).

### Quality assessment

The quality of included studies was independently evaluated by two investigators (M.A.E. and A.K.A.). Disagreements were resolved by consensus. The Cochrane ROBINS-I tool was used for observational studies,^9^ and the Cochrane Risk of Bias 2.0 (RoB 2) tool^10^ was applied for randomized controlled trials.

### Statistical analysis

All analyses were performed using R Studio (version 4.4.3, R Foundation for Statistical Computing). To account for variation in follow-up durations among studies, incidence rate ratios (IRR) with 95% confidence intervals (CI) were pooled using a random-effects model. Heterogeneity was assessed using I^2^ statistic, with thresholds interpreted as low, moderate, and high heterogeneity as 0%-49%, 50%-75%, and >75%, respectively. Heterogeneity was considered significant when the Chi-square *P*-value was <.10. Sensitivity analyses were performed by sequentially excluding each study to assess the influence of individual studies on pooled estimates. Statistical significance was defined as *P* < .05. For time-to-event outcomes, we extracted data from published Kaplan–Meier curves. Kaplan–Meier curves were digitized using WebPlotDigitizer (v4.7), and IPD were reconstructed following Guyot et al.^11^ The reconstructed IPD was additionally cross-validated by comparing reconstructed versus reported ones. Digitized survival data, together with the total number of patients at risk, events, and patients at risk at specified time points, were used to reconstruct individual patient data (IPD) and estimate pooled hazard ratios (HRs). Cox proportional hazards models were fitted to reconstructed IPD to estimate study-level HRs. Schoenfeld residuals were used to test the proportional hazards (PH) assumption; when violated, time-varying effects were examined. A random-effects model was used to pool estimates. Publication bias was evaluated for outcomes reported in ≥10 studies using visual inspection of funnel plots and Egger’s regression test.

## Results

### Literature search

Our search identified 1589 records. After removal of duplicates and title/abstract screening, 48 full-text articles were assessed for eligibility. Of these, 17 studies met the inclusion criteria and were included in the analysis^6^^,^^7^^,^^12–26^ (**Figure S1**).

### Characteristics of the included studies

Seventeen studies comprising a total of 7338 participants were included, of whom 2896 underwent AVR and 4656 received conservative management. Among the included studies, 13 were observational and 4 were RCTs. The mean age across studies ranged from 63 to 85 years. SAVR was the predominant intervention. A summary of the studies and patient characteristics is presented in **Table 1** and **Table S2**.

**Table 1.** Baseline Characteristics of Patients in the Included Studies

Author, Year	Sample size		Follow-up period (Years)		Age, Mean (SD)		Sex (Male), N		BMI, Mean (SD)		Cardiovascular history, N											
											Hypertension		CAD		Diabetes		Hyperlipidemia		Atrial Fibrillation		Chronic Lung Disease	
	Intervention	Control	Intervention	Control	Intervention	Control	Intervention	Control	Intervention	Control	Intervention	Control	Intervention	Control	Intervention	Control	Intervention	Control	Intervention	Control	Intervention	Control
Pai 2006	99	239	11		67(15)	73(14)	43	129	NA	NA	52	65	16	50	17	19	NA	NA	NA	NA	8	21
Kang 2010	102	96	5		63(11)	63(12)	55	44	23.9 (2.8)	24.1 (3.5)	37	39	NA	NA	10	10	31	37	NA	NA	NA	NA
Le Tourneau 2010	160	514	5		69(10)	72(11)	91	322	26(4)	27(6)	64	229	NA	NA	8	65	NA	NA	6	25	NA	NA
Taniguchi 2015	291	1517	5		71.6(8.7)	77.8(9.4)	126	604	22.1 (3.3)	21.9 (3.9)	188	1060	28	356	59	375	116	532	39	299	27	134
Masri 2016	533		6.9		66(13)		415		28(5)		369		NA		84		365		59		NA	
Bohbot 2018	192	247	5		73 (10)	73 (11)	NA	NA	NA	NA	NA	NA	NA	NA	NA	NA	NA	NA				
Kim 2019	221	247	5		61(12.3)	67.1(13.1)	110	126	24.6(3.2)	23.6(3.2)	92	122	32	17	37	66	59	61	19	34	24	34
Campo 2019	104	161	5		68.1(11.7)	73(12.6)	73	95	NA	NA	75	117	37	66	13	34	NA	NA	NA	NA	16	17
Miura 2019	210	360	3.9		74.6(8.8)	80.9(8.3)	94	142	22.8(3.2)	21.6(3.4)	144	251	75	155	60	95	96	133	22	39	37	86
Kang 2020	73	72	4		65(7.8)	63.4(10.7)	37	34	24.7(3.4)	24(2.6)	40	39	5	1	13	7	41	41	3	6	NA	NA
Banovic 2021	78	79	2.9		60.75(12.6)	68.75(7.6)	46	44	27.2(4.3)	27.4(4.6)	69	70	NA	NA	14	23	31	28	NA	NA	NA	NA
Celik 2021	46	13	8.9		66.5(10.6)	74.1(8.9)	32	12	26.9(3.7)	27.5(3.9)	24	5	4	0	6	6	21	8	2	2	4	2
Loganath 2024	113	111	3.5		75 (68-79)^a^	76 (68-80)^a^	82	79	27.2 (24.4-31.1)^a^	27.8 (24.8-31.1)^a^	76	70	5	8	15	26	55	56	NA	NA	NA	NA
Heuvelman 2012	22	37	2		69.9 (14.8)		47		NA	NA	49		5		12		28		NA	NA	6	
Takeji 2025	206	206	3		79.1 (7.9)	80.0 (8.1)	78	75	22.8 (3.5)	23.0 (3.6)	168	177	16	33	42	55	110	129	20	23	7	7
Takeji 2019	278	278	809 (: 736-1118)^a^ days	1155 (903-1590)^a^ days	84.6 (5.7)	85.1 (7.0)	81	81	21.4 (3.5)	21.8 (3.9)	211	211	114	100	81	61	140	105	31	55	72	29
Généreux 2025	455	446	3.8		76.0 ± 6.0	75.6 ± 6.0	324	299	28.4 (4.6)	28.6 (4.8)	369	365	133	113	119	114	375	347	71	59	13	15
Merhi 2021	62	76	1		73.3(6.5)	75.0(5.0)	49	52	NA	NA	48	60	NA	NA	17	19	NA	NA				

a Data are presented as Median (Inter quartile range).

Abbreviations: NA = Not Available; SAVR = Surgical Aortic Valve Replacement; TAVR = Transcatheter Aortic Valve Replacement; CS = Conservative Strategy; CAD = Coronary Artery Disease; AVR = Aortic Valve Replacement.

### Risk of bias assessment

The RCTs showed low risk of bias across all domains when assessed using RoB 2, indicating overall low risk of bias among included trials (**Figure S2A**). The observational studies were assessed using the ROBINS-I tool and demonstrated (**Figure S2B, C**).

### Outcomes

#### All-cause mortality

**Figure 1.**
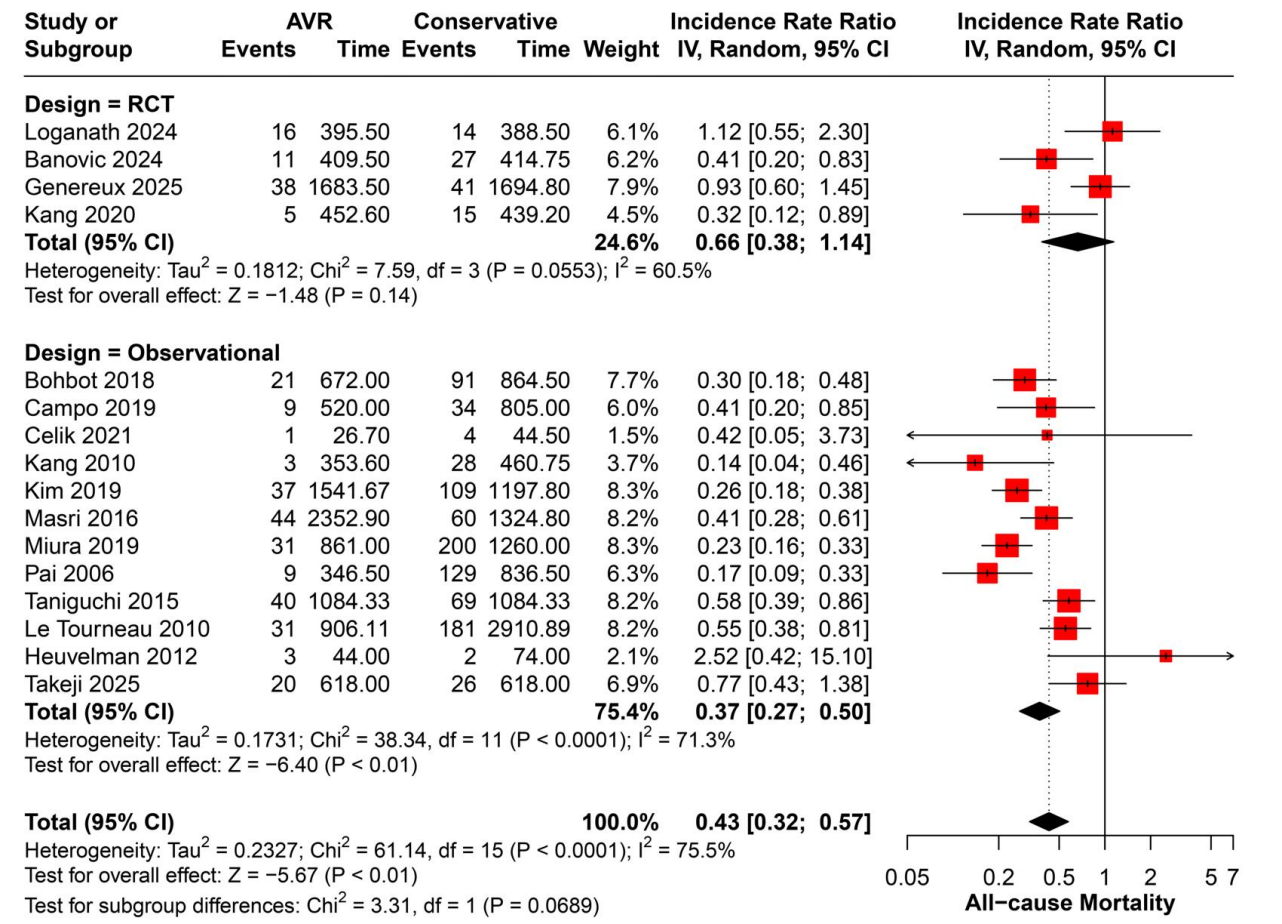
Forest Plot of All-Cause Mortality

Sixteen studies reported all-cause mortality. AVR was associated with a significantly lower risk compared with conservative management (IRR = 0.43, 95% CI 0.32-0.57; *P* < .001), with moderate heterogeneity (I^2^ = 75%, *P* < .001). In subgroup analysis, the effect was mainly driven by observational studies (IRR = 0.37, 95% CI 0.27-0.50; *P* < .001), whereas the RCT subgroup did not reach statistical significance (IRR = 0.66, 95% CI 0.38-1.14; *P* = .14) (**Figure 1**).

#### Cardiovascular mortality

**Figure 2.**
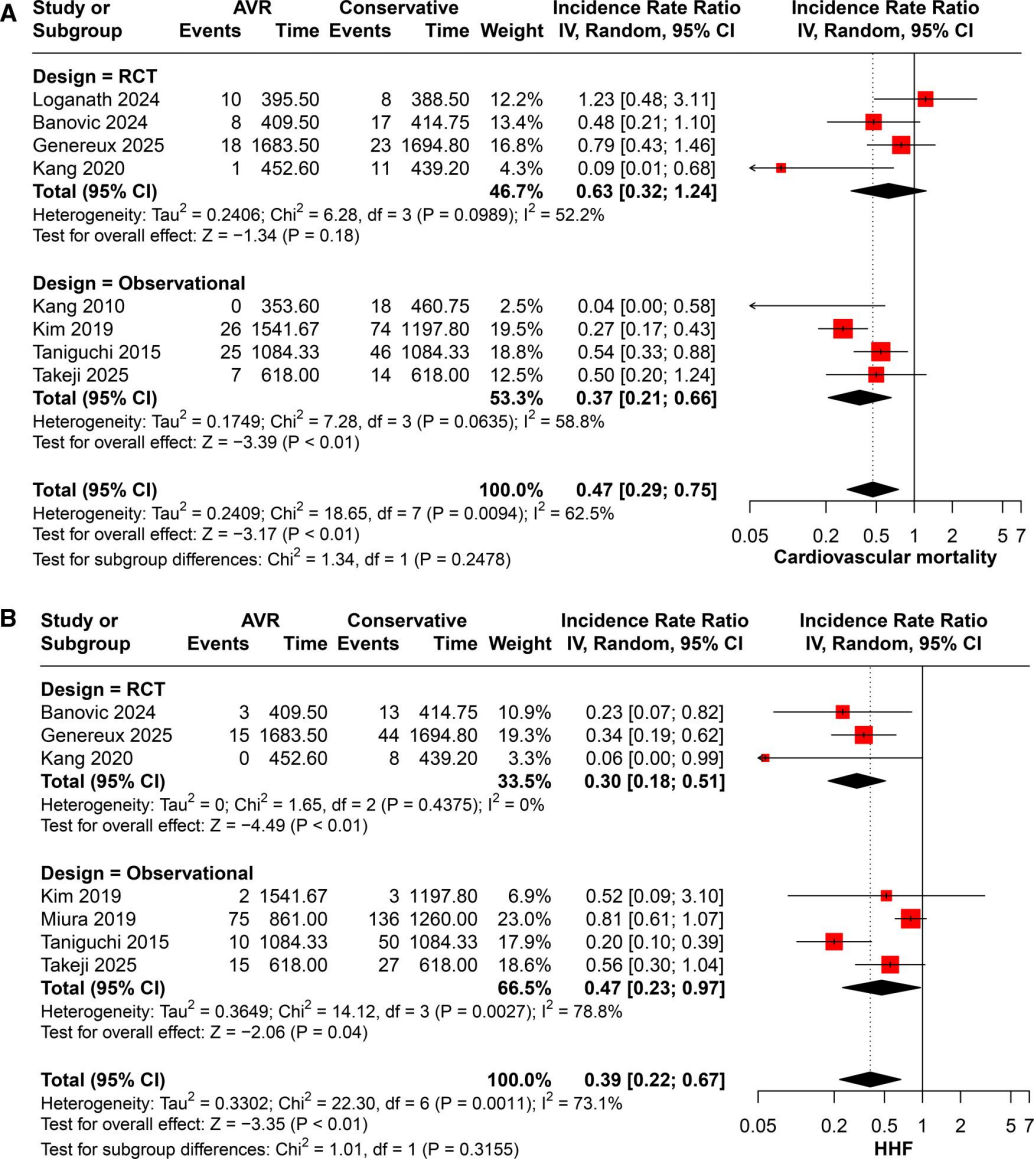
Forest Plots of (A) Cardiovascular Mortality and (B) Hospitalization for Heart Failure

For cardiovascular mortality, the AVR group was associated with statistically significant lower risk (IRR = 0.47, 95% CI 0.29-0.75; *P* < .01), with moderate heterogeneity (I^2^ = 62%, *P* = .01). This effect was predominantly derived from observational studies (IRR = 0.37, 95% CI 0.21-0.66; *P* < .01), while the RCT subgroup showed a nonsignificant trend (IRR = 0.63, 95% CI 0.32-1.24; *P* = .18) (**Figure 2A**).

#### Heart failure hospitalization (HHF)

AVR significantly reduced the risk of HHF (IRR = 0.39, 95% CI 0.22-0.67; *P* < .01), with moderate heterogeneity (I^2^ = 73%, *P* = .01). Both observational (IRR = 0.47, 95% CI 0.23-0.79; *P* = .04) and RCT (IRR = 0.30, 95% CI 0.18-0.51; *P* < .01) subgroups showed consistent results (**Figure 2B**).

#### Sudden cardiac death

**Figure 3.**
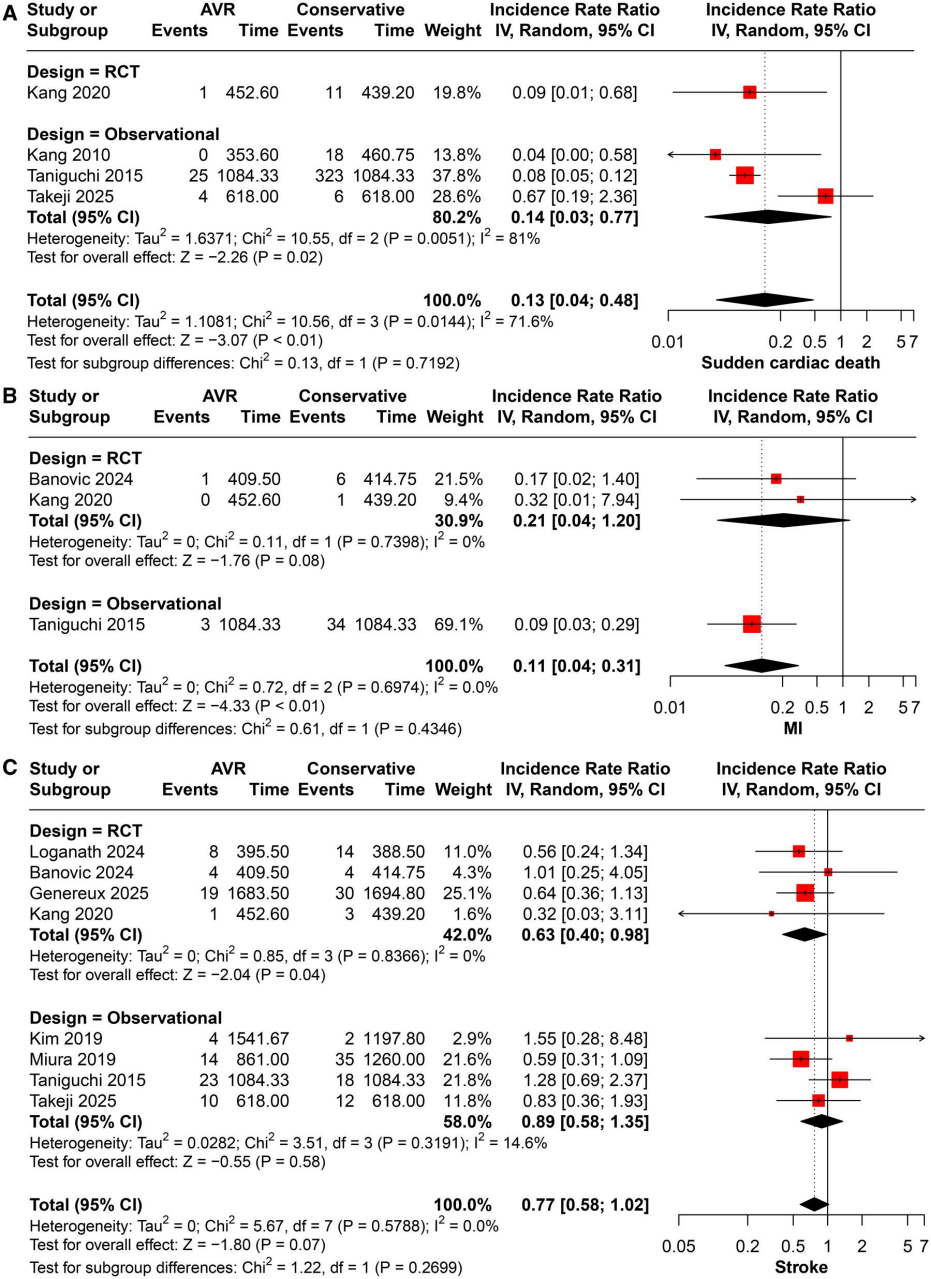
Forest Plots for the Analysis of (A) Sudden Cardiac Death and (B) myocardial Infarction (MI)

The AVR group was associated with statistically significant lower sudden cardiac death (IRR = 0.13, 95% CI 0.04-0.48, *P* < .01), with moderate heterogeneity (*P* < .001, I2 = 71%). Subgrouping showed that this was derived via an RCT (IRR = 0.09, 95% CI 0.01-0.68), and the observational subgroup was statistically significant (IRR = 0.14, 95% CI 0.04-0.48, *P* = .02) (**Figure 3A**).

#### Cerebrovascular and cardiovascular events

AVR significantly reduced the risk of myocardial infarction (MI) (IRR = 0.11, 95% CI 0.04-0.31; *P* = .03), with low heterogeneity (I^2^ = 0%, *P* = .67). The effect was primarily observed in observational studies (IRR = 0.09, 95% CI 0.03-0.29), while RCTs showed a nonsignificant trend (IRR = 0.21, 95% CI 0.04-1.20; *P* = .08) (**Figure 3B**).

For Stroke, AVR was associated with no significantly different risk of stroke (IRR = 0.77, 95% CI 0.58-1.02; *P* = .07), with low heterogeneity (I^2^ = 0%, *P* = .57). Subgrouping showed that the RCT subgroup was associated with significantly different lower risk (IRR = 0.63, 95% CI 0.40-0.98), whereas the observational subgroup did not reach statistical significance (IRR = 0.89, 95% CI 0.58-1.35; *P* = .58) (**Figure 3C**).

### Sensitivity analyses

A series of leave-one-out sensitivity analyses was conducted to evaluate the influence of individual studies. The significance and magnitude of the pooled effects for all-cause mortality, cardiovascular mortality, and HHF remained stable regardless of study exclusion (**Figure S3A-C**). Conversely, results for sudden cardiac death, MI, and stroke were more sensitive to individual study removal (**Figure S3D-F**).

#### Publication bias

**Figure 4.**
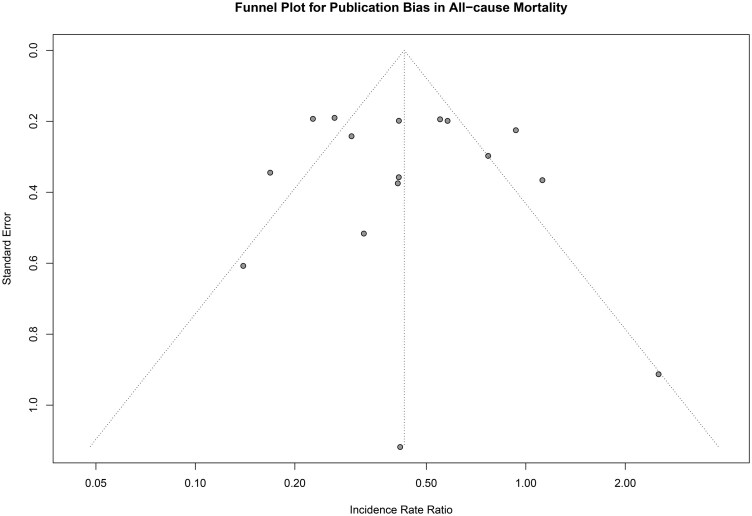
Forest Plot for the Analysis of Stroke

Assessment of publication bias for the primary outcome, all-cause mortality, was performed as it included more than ten studies. Visual inspection of the funnel plot showed no asymmetrical distribution of effect estimates, suggesting no significant small-study effects. This was further supported by Egger’s regression test, which demonstrated no evidence of publication bias (*P* = .715) (**Figure 4**).

#### Reconstructed time-to-event analysis

**Figure 5.**
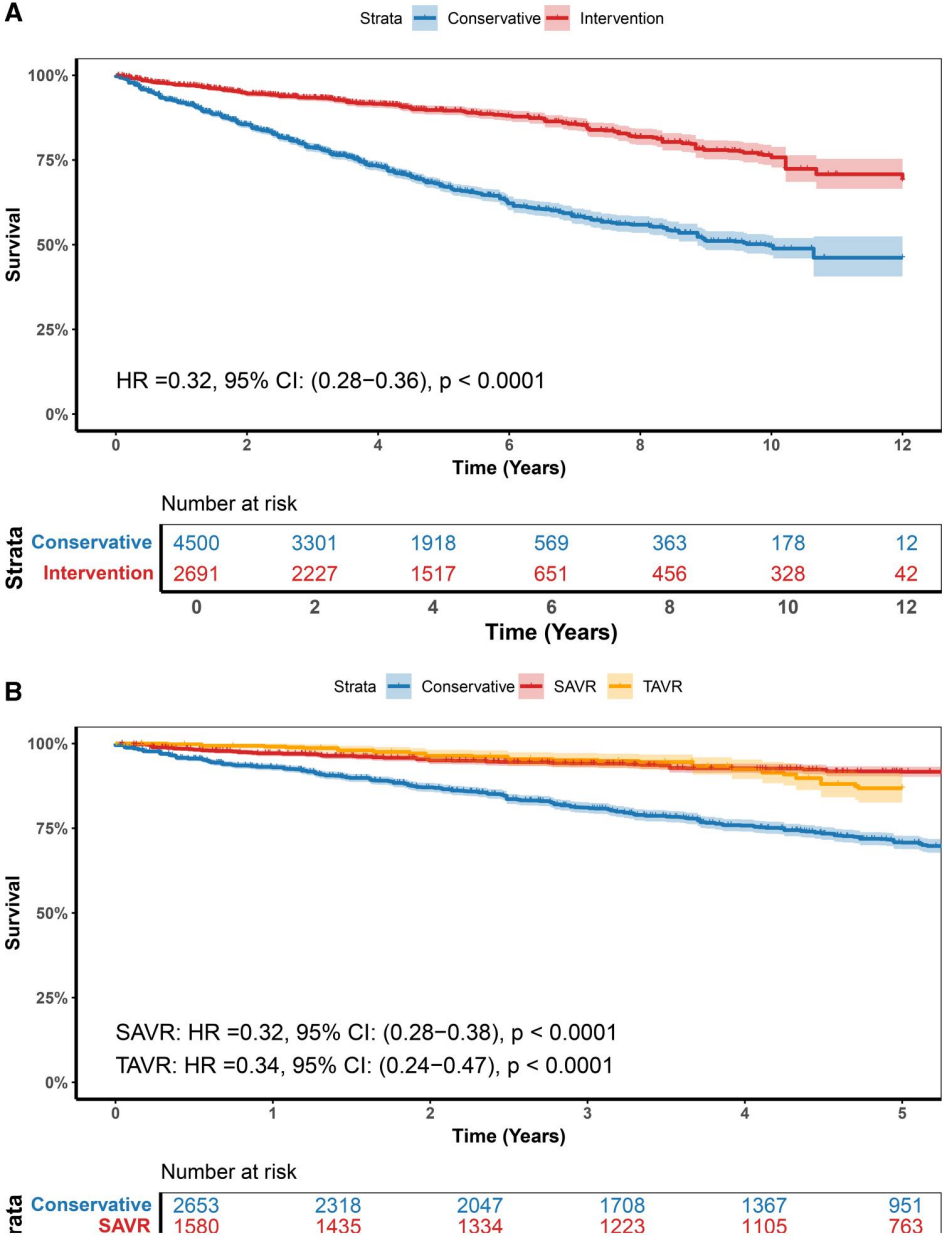
Funnel Plots regarding All-Cause Mortality

In the reconstructed Kaplan–Meier survival analysis over a 12-year follow-up, early AVR was associated with significantly improved long-term survival compared with conservative management (HR = 0.32; 95% CI, 0.28-0.36; *P* < .0001) (**Figure 5A**). Assessment of the proportional hazards (PH) assumption using Schoenfeld residuals demonstrated a significant violation (*P* = .0029), indicating time-varying treatment effects (**Figure S4**). Time-dependent hazard modelling revealed that although the hazard ratio remained consistently below 1 throughout follow-up, the magnitude of benefit progressively attenuated over time (**Figure S5**). When stratified by intervention type, both SAVR (HR = 0.32; 95% CI, 0.28-0.38; *P* < .0001) and TAVR (HR = 0.34; 95% CI, 0.24-0.47; *P* < .0001) were associated with superior survival relative to conservative management (**Figure 5B**). However, pooled data from RCTs didn’t reach statistical significance in long-term mortality between AVR and conservative management (HR = 0.79; 95% CI, 0.57-1.08; *P* = .13) (**Figure S6**).

## Discussion

Our study is the first meta-analysis that pools studies from all eligible high-quality RCTs and observational studies to synthesize evidence on the benefits of early AVR, with stratification of surgical or transcatheter approaches, over conservative treatment. In addition, we were able to analyze data from a large sample size. Moreover, we incorporated Kaplan–Meier survival analysis, which allowed us to assess time-to-event data and provide a more comprehensive understanding of long-term survival benefits associated with early AVR. We found that early aortic valve AVR, whether through surgical aortic valve replacement or transcatheter aortic valve replacement, was associated with significantly lower risk for all-cause and cardiovascular mortality compared to conservative management. Specifically, AVR was associated with a 57% reduction in all-cause mortality and a 53% reduction in cardiovascular mortality compared with conservative management.

Previous meta-analyses did not demonstrate that early AVR is beneficial in patients with asymptomatic AS. A meta-analysis by Lim et al. evaluating 1300 patients found no significant survival advantage for early AVR over conservative management concerning all-cause mortality, cardiac mortality, and sudden cardiac death. The discrepancy in findings can be attributed to the smaller sample size in Lim et al. and the considerable heterogeneity in all-cause and cardiac mortality observed in their study.^27^ Similarly, Kumar et al. reported no difference in sudden cardiac arrest, stroke, or MI, although their study included only 3814 patients.^28^ Their study also had a limited number of RCTs. The availability of recent RCTs has demonstrated changes in these outcomes. The RECOVERY trial demonstrated that early surgical AVR reduced perioperative mortality to approximately 1% and completely prevented sudden cardiac death.^15^ Advances in surgical techniques and expertise have lowered the risks associated with AVR, making early intervention a more viable and safer option for patients with asymptomatic severe AS.

Early AVR is increasingly supported due to reduced surgical risks from advances in technology and perioperative care, particularly in SAVR, as shown in the RECOVERY trial and studies comparing SAVR to TAVR in low-risk patients.^15^ Conservative management lacks systematic follow-up and often misclassifies patients as asymptomatic due to the absence of stress testing, leading to delayed intervention, increased risk of sudden death, and worse outcomes.^28^ The RECOVERY trial highlighted these issues but was limited by its focus on very severe AS.^15^ The AVATAR trial addressed these gaps by including asymptomatic patients with severe (but not critical) AS and preserved LV function, using stress testing to confirm eligibility. It demonstrated that early SAVR significantly improved a composite end-point of death, MI, stroke, and HF hospitalization, supporting early AVR in well-selected patients.^18^ Stroke itself has been reported to not being affected which is consistent with our findings. Subgroup analysis using RCTs data showed lower risk with early intervention, which can be attributed to the prevention of the progression to symptomatic disease, heart failure, and atrial fibrillation, all of which are associated with increased thromboembolic risk.

The EARLY TAVR trial supports early intervention in asymptomatic severe AS, showing that TAVR significantly reduced the primary end-point, including stroke and unplanned cardiovascular hospitalizations, compared to clinical surveillance, despite similar overall mortality. During a median follow-up of 3.8 years, 87% of the surveillance group eventually required aortic valve replacement, indicating inevitable disease progression and the risks of delayed treatment. The study also found no significant difference in procedural complications between groups, challenging the idea that early intervention increases risk. However, its focus on low surgical risk patients limits generalizability to higher-risk populations.^6^

Despite early AVR showing better outcomes in stroke, myocardial infarction, and sudden cardiac death across some studies, the choice between SAVR and TAVR depends on individual factors such as comorbidities, anatomy, and surgical risk. Differences in studies’ patient selection criteria may explain the apparent superiority of SAVR in secondary outcomes.^6^^,^^7^^,^^12–24^^,^^29–31^ A multidisciplinary heart team approach is essential for personalized decision-making, which is in align with the most recent recommendations of the 2025 ESC/EACTS valve guidelines^32^ recommend conservative management with surveillance for most asymptomatic patients with severe aortic stenosis and preserved left ventricular function. Guidelines acknowledge as well that early aortic valve replacement may reduce mortality, heart failure hospitalization, and adverse cardiovascular events in select high-risk patients with very severe AS, rapid progression, or myocardial fibrosis. Thus, the guidelines advocate for individualized decision-making through a multidisciplinary heart team, considering clinical risk factors, imaging findings, and patient preferences. Early AVR is not universally recommended but may be considered in high-risk patients where benefits outweigh procedural risks. However, our findings support a proactive strategy to prevent irreversible myocardial remodelling. On the other side, we believe that minimally invasive SAVR techniques now with its proven safety profiles comparable to TAVR with durable long-term outcomes might be an valid option as well for these patients.^33^^,^^34^ Regular monitoring with echocardiography and stress testing is vital in asymptomatic patients to guide timely intervention. Future approaches may combine imaging, biomarkers, and clinical data to personalize treatment timing. Trials like EVoLVeD and EASY-AS are expected to improve risk stratification using markers of early left ventricular decompensation^35^^,^^36^

### Strengths and limitations

Our study has some limitations. First, baseline differences existed among the included study populations, particularly regarding the high mean age of our included patients and the relatively small number of studies using TAVR as an intervention arm. We addressed this using a random-effects model and the inverse variance method of analysis. Second, heterogeneity was present in some analyses, but sensitivity analysis helped mitigate its impact. Many of our secondary outcome’s results are reported without correction which can increase the risk of multiplicity and false positive findings; thus, results shall be interpreted in caution. Additionally, due to the lack of individual patient data, we could not exclude patients with low left ventricular ejection fraction or those requiring coronary artery bypass grafting from our analyses. Another limitation is the variability in the definition of severe asymptomatic AS across studies, with some using LVEF, mean aortic gradient, or transaortic velocity as criteria. Furthermore, the type of aortic valve replacement (surgical or transcatheter) was not consistently declared in most included studies, preventing us from conducting a direct comparison of SAVR versus TAVR. Given these limitations, more RCTs are needed, particularly those directly comparing SAVR and TAVR in asymptomatic severe AS, to further refine patient selection and optimize treatment strategies.

### Conclusion

In conclusion, our findings reinforce the clinical benefit of early AVR in asymptomatic severe AS, particularly with SAVR, which was associated with significant reductions in mortality and adverse cardiovascular events. Although TAVR demonstrated improved all-cause and cardiovascular survival, its role in reducing non-cardiovascular mortality and other adverse outcomes remains inconclusive. Given the evolving landscape of AS management, future research should focus on refining patient selection criteria, optimizing procedural safety, and assessing long-term outcomes to guide evidence-based decision-making in terms of the optimal approach (TAVR vs SAVR). Heart team discussion is always warranted to guide the optimal individualized approach.

## Supplementary Material

ivaf308_Supplementary_Data

## Data Availability

All data are available and attached.

## DECLARATIONS

N/A.
