# Supplementary material for: Surgical vs Transcatheter Aortic Valve Replacement for Asymptomatic Severe Aortic Stenosis- an Updated Meta-Analysis of Reconstructed Individual Patient Data
Source: Interdiscip Cardiovasc Thorac Surg. 2025 Dec 24;41(1):ivaf308. doi: 10.1093/icvts/ivaf308 (PMC12798724; doi:10.1093/icvts/ivaf308)

**Supplementary Table 1:** Detailed search strategy of different databases.

| PubMed | ("Aortic Valve Stenosis"[MeSH] OR "Aortic Valve Stenosis"[tiab] OR "aortic stenosis"[tiab] OR "aortic valve stenosis"[tiab] OR "severe aortic stenosis"[tiab] OR "critical aortic stenosis"[tiab]) AND  AND  (("Transcatheter Aortic Valve Replacement"[MeSH] OR "Transcatheter Aortic Valve Replacement"[tiab] OR "transcatheter aortic valve replacement"[tiab] OR TAVR[tiab] OR TAVI[tiab] OR "transcatheter aortic valve implantation"[tiab])  OR ("Aortic Valve Replacement"[MeSH] OR "aortic valve replacement"[tiab] OR AVR[tiab] OR "surgical aortic valve replacement"[tiab] OR SAVR[tiab] OR "surgical valve replacement"[tiab]) OR ("early surgery"[tiab] OR "early replacement"[tiab] OR "early AVR"[tiab] OR "early TAVR"[tiab] OR "early valve replacement"[tiab] OR immediate[tiab] OR "early intervention"[tiab]))  AND  (conservative[tiab] OR "conservative management"[tiab] OR "watchful waiting"[tiab] OR "clinical surveillance"[tiab] OR "medical management"[tiab] OR "no intervention"[tiab] OR "watch and wait"[tiab] OR "active surveillance"[tiab]) | N= 429 |
| --- | --- | --- |
| Cochrane CENTRAL | "Aortic Valve Stenosis" OR "aortic stenosis" OR "aortic valve stenosis" OR "severe aortic stenosis" OR "critical aortic stenosis" in Title Abstract Keyword AND "Transcatheter Aortic Valve Replacement" OR "Transcatheter Aortic Valve Replacement" OR "transcatheter aortic valve replacement" OR TAVR OR TAVI OR "transcatheter aortic valve implantation" OR "Aortic Valve Replacement" OR "aortic valve replacement" OR AVR OR "surgical aortic valve replacement" OR SAVR OR "surgical valve replacement" OR "early surgery" OR "early replacement" OR "early AVR" OR "early TAVR" OR "early valve replacement" OR immediate OR "early intervention" in Title Abstract Keyword AND conservative OR "conservative management" OR "watchful waiting" OR "clinical surveillance" OR "medical management" OR "no intervention" OR "watch and wait" OR "active surveillance" in Title Abstract Keyword | N=64 |
| Web of science (WOS) | "Aortic Valve Stenosis" OR "aortic stenosis" OR "aortic valve stenosis" OR "severe aortic stenosis" OR "critical aortic stenosis" (All Fields) AND "Transcatheter Aortic Valve Replacement" OR "Transcatheter Aortic Valve Replacement" OR "transcatheter aortic valve replacement" OR TAVR OR TAVI OR "transcatheter aortic valve implantation" OR "Aortic Valve Replacement" OR "aortic valve replacement" OR AVR OR "surgical aortic valve replacement" OR SAVR OR "surgical valve replacement" OR "early surgery" OR "early replacement" OR "early AVR" OR "early TAVR" OR "early valve replacement" OR immediate OR "early intervention" (All Fields) AND conservative OR "conservative management" OR "watchful waiting" OR "clinical surveillance" OR "medical management" OR "no intervention" OR "watch and wait" OR "active surveillance" (All Fields) | N=386 |
| Scopus | ( TITLE-ABS-KEY ( "Aortic Valve Stenosis" OR "aortic stenosis" OR "aortic valve stenosis" OR "severe aortic stenosis" OR "critical aortic stenosis" ) AND TITLE-ABS-KEY ( "Transcatheter Aortic Valve Replacement" OR "Transcatheter Aortic Valve Replacement" OR "transcatheter aortic valve replacement" OR TAVR OR TAVI OR "transcatheter aortic valve implantation" OR "Aortic Valve Replacement" OR "aortic valve replacement" OR AVR OR "surgical aortic valve replacement" OR SAVR OR "surgical valve replacement" OR "early surgery" OR "early replacement" OR "early AVR" OR "early TAVR" OR "early valve replacement" OR immediate OR "early intervention" ) AND TITLE-ABS-KEY ( conservative OR "conservative management" OR "watchful waiting" OR "clinical surveillance" OR "medical management" OR "no intervention" OR "watch and wait" OR "active surveillance" ) ) | N= 710 |
| Total= 1589 | | |

| **Table S2.** Summary of the included studies | | | | | | | | | | | |
| --- | --- | --- | --- | --- | --- | --- | --- | --- | --- | --- | --- |
| **Study ID** | **Design** | **Setting** | **Sample Size** | **Study Duration (Year)** | **Inclusion criteria** | **Intervention** | | **Comparator** | | **Primary Outcomes** |  |
|  |  |  |  |  |  | **No. of patients** | **Approach** | **No. of patients** | **Approach** |  |  |
| Loganath 2024 | RCT | UK | 224 | 2017-2022 | Age ≥18 y; Vmax ≥ 4.0 m/s or iAVA < 0.6 cm²/m² and Vmax ≥ 3.5 m/s; midwall LGE on CMR; asymptomatic (no AVR indication); LVEF ≥ 50%; no stress test reported. | 113 | TAVI/ SAVR | 111 | CS | Composite of all-cause death or unplanned aortic stenosis–related hospitalization. |  |
| Banovic 2024 | RCT | Europe | 157 | 2015-2023 | Age ≥18 y; AVA ≤ 1.0 cm² or iAVA ≤ 0.6 cm²/m² at rest and Vmax > 4.0 m/s or MG ≥ 40 mm Hg; asymptomatic; STS < 8%; LVEF ≥ 50%; low-level stress test (100%). | 78 | SAVR | 97 | CS | Composite of all-cause death, acute myocardial infarction, stroke, or unplanned hospitalization for heart failure (HF). |  |
| Genereux 2025 | RCT | USA, Canda | 901 | 2017-2021 | Age ≥65 y; AVA ≤ 1.0 cm² or iAVA ≤ 0.6 cm²/m² and Vmax ≥ 4.0 m/s or MG ≥ 40 mm Hg; asymptomatic (confirmed by exercise testing); STS ≤ 10; LVEF ≥ 50%; low-level stress test (90.6%). | 455 | TAVI | 446 | CS | Composite of death from any cause, stroke, or unplanned hospitalization for cardiovascular causes. |  |
| Kang 2020 | RCT | Korea | 145 | 2010-2015 | Age 20–80 y; AVA ≤ 0.75 cm² and Vmax ≥ 4.5 m/s or MG ≥ 50 mm Hg; asymptomatic; eligible for early surgery; LVEF ≥ 50%; low-level stress test (17%). | 73 | SAVR | 72 | CS | Operative mortality or cardiovascular death during follow-up. |  |
| Bohbot 2018 | Observational | France, Belgium | 439 | 2000-2015 | Isolated high-gradient severe AS (MG > 40 mm Hg); LVEF > 50%; asymptomatic; EuroSCORE II < 4%. | 192 | SAVR | 247 | CS | Death during follow-up. |  |
| Campo 2019 | Observational | USA | 265 | 2005-2013 | Severe AS defined as AVA ≤ 1.0 cm², MG ≥ 40 mm Hg, or Vmax ≥ 4.0 m/s. | 104 | SAVR | 161 | CS | Death during follow-up. |  |
| Celik 2021 | Observational | Netherlands | 8 | 2006-2009 | Severe AS defined as Vmax > 4.0 m/s or AVA < 1.0 cm². | 3 | TAVI/ SAVR | 5 | CS | All-cause mortality. |  |
| Kang 2010 | Observational | Korea | 197 | 1996-2006 | Severe AS with AVA ≤ 0.75 cm² and Vmax ≥ 4.5 m/s or MG ≥ 50 mm Hg. | 102 | SAVR | 95 | CS | Composite of operative mortality and cardiac death during follow-up. |  |
| Kim 2019 | Observational | Korea | 468 | 2000-2015 | Severe AS (AVA ≤ 1.0 cm², Vmax ≥ 4.0 m/s, or MG ≥ 40 mm Hg). | 221 | SAVR | 247 | CS | Death during follow-up. |  |
| Masri 2016 | Observational | USA | 533 | 2001-2012 | Severe AS (iAVA ≤ 0.6 cm²/m²) undergoing exercise treadmill echocardiography. | 341 | SAVR | 192 | CS | Death during follow-up. |  |
| Miura 2019 | Observational | Japan | 570 | 2009-2012 | Severe AS based on guidelines: AVA ≤ 1.0 cm² (by TTE). | 360 | SAVR | 210 | CS | All-cause mortality and cardiovascular events. |  |
| Pai 2006 | Observational | USA | 338 | 1993-2003 | Severe AS defined as AVA ≤ 0.8 cm². | 99 | SAVR | 239 | CS | Death during follow-up. |  |
| Taniguchi 2015 | Observational | Japan | 1808 | 2003-2011 | Severe AS (Vmax > 4.0 m/s, MG > 40 mm Hg, or AVA < 1.0 cm²). | 291 | TAVI/ SAVR | 1517 | CS | All-cause death and hospitalization for heart failure (HF). |  |
| Le Tourneau 2010 | Observational | USA | 674 | 1984-1995 | Age ≥40 y; isolated, asymptomatic severe AS (Vmax > 4.0 m/s). | 160 | SAVR | 514 | CS | Operative mortality within 1 year, long-term survival (10-year), and cardiac death. |  |
| Heuvelman 2012 | Observational | Netherlands | 59 | 2006-2009 | Age ≥18 y; AVA ≤ 1.0 cm², Vmax ≥ 4.0 m/s, or VTI ratio ≥ 4.0. | 22 | TAVI/ SAVR | 37 | CS | Death during follow-up. |  |
| Takeji 2025 | Observational | Japan | 414 | 2018-2020 | Severe AS newly diagnosed, meeting ≥1 criterion: Vmax > 4.0 m/s, MG > 40 mm Hg, or AVA < 1.0 cm². | 206 | TAVI/ SAVR | 206 | CS | Composite of all-cause death, stroke, or hospitalization for heart failure (HF). |  |
| Merhi 2021 | Observational | USA | 138 | 2021 | Asymptomatic severe AS with (1) MG ≥ 60 mm Hg or Vmax ≥ 5.0 m/s; (2) LVEF < 50%; or (3) reduced ETT response. | 76 | TAVR | 62 | SAVR | Composite of all-cause mortality or disabling stroke at 12 months. |  |
| AS = aortic stenosis; HF = heart failure; MI = myocardial infarction; AVR = aortic valve replacement; LVEF = left ventricular ejection fraction; STS = Society of Thoracic Surgeons risk score; Vmax = peak transaortic velocity; AVA = aortic valve area; iAVA = indexed aortic valve area; MG = mean gradient; TTE = transthoracic echocardiography; ETT = exercise treadmill test; LGE = late gadolinium enhancement; CMR = cardiac magnetic resonance; EuroSCORE = European System for Cardiac Operative Risk Evaluation. | | | | | | | | | | | |

***Table S3.*** Summary of Pooled Outcomes Comparing Aortic Valve Replacement (AVR) vs. Conservative Management.

| **Outcome** | **No. of Studies** | **Effect Size (95% CI)** | **p-value** | **Heterogeneity (I², p)** | **Subgroup Findings** |
| --- | --- | --- | --- | --- | --- |
| **All-cause mortality** | 16 | IRR = 0.43 (0.32–0.57) | < 0.001 | 75%, < 0.001 | Observational: 0.37 (0.27–0.50), p<0.001; RCTs: 0.66 (0.38–1.14), p=0.14 |
| **Cardiovascular mortality** | 9 | IRR = 0.47 (0.29–0.75) | < 0.01 | 62%, 0.01 | Observational: 0.37 (0.21–0.66), p<0.01; RCTs: 0.63 (0.32–1.24), p=0.18 |
| **Heart failure hospitalization (HHF)** | 7 | IRR = 0.39 (0.22–0.67) | < 0.01 | 73%, 0.01 | \|  \| \| --- \|  \| Observational: 0.47 (0.23–0.79), p=0.04; RCTs: 0.30 (0.18–0.51), p<0.01 \| \| --- \| |
| **Sudden cardiac death** | 6 | IRR = 0.13 (0.04–0.48) | < 0.01 | 71%, < 0.001 | Observational: 0.14 (0.04–0.48), p=0.02; RCTs: 0.09 (0.01–0.68) |
| **Myocardial infarction** | 3 | IRR = 0.11 (0.04–0.31) | 0.03 | 0%, 0.67 | Observational: 0.09 (0.03–0.29); RCTs: 0.21 (0.04–1.20), p=0.08 |
| **Stroke** | 9 | IRR = 0.77 (0.58–1.02) | 0.07 | 0%, 0.57 | \|  \| \| --- \|  \| RCTs: 0.63 (0.40–0.98); Observational: 0.89 (0.58–1.35), p=0.58 \| \| --- \| |
| **SAVR vs. TAVR (Mehri et al.)** | 1 | — | — | — | ↑ Bleeding & PPM with TAVR; ↑ AF with SAVR |

NS: Non-significant, Obs: observational, RCT: Randomized controlled trial, AVR: Aortic valve replacement

***Figure S1.*** PRISMA flow diagram of the literature search.

***Figure S2.*** Risk of bias assessment of the included studies using (A) ROB-2 and (B) ROBIN-I tools.

Quality check: For randomized controlled trials (RCTs), Quality assessment using the Cochrane Risk of Bias 2 tool revealed that all four included RCTs (Généreux et al. 2025, Loganath et al. 2024, Banovic et al. 2021, and Kang et al. 2020) demonstrated low risk of bias across all five domains: randomization process, deviations from intended interventions, missing outcome data, outcome measurement, and selection of reported results (Figure S2A). Overall, the methodological quality of included studies was high. Moreover, The methodological quality of the 12 included observational studies was assessed using the Risk Of Bias In Non-randomized Studies - of Interventions (ROBINS-I) tool across seven bias domains (Figure S2B and C). Overall, the majority of included studies demonstrated low to moderate risk of bias. Five studies (Kang et al. 2010, Kim et al. 2019, Le Tourneau et al. 2010, Heuvelman et al. 2012, and Takeji et al. 2025) were rated as low risk across all domains with an overall low risk judgment. Five studies (Bohbot et al. 2018, Celik et al. 2021, Masri et al. 2016, Miura et al. 2019, and Taniguchi et al. 2015) received an overall moderate risk of bias rating, primarily due to concerns regarding bias from missing data (D5), bias in measurement of outcomes (D6), or bias in selection of participants (D2). One study (Campo et al. 2019) had unclear risk in the domain of bias due to selection of participants (D2) but was overall rated as low risk. Only one study (Pai et al. 2006) was rated as overall serious risk of bias due to serious concerns in bias from classification of interventions (D3) and bias in measurement of outcomes (D6). The summary assessment across all studies (Figure S2C) demonstrated that bias due to confounding (D1) was predominantly low risk across studies. However, several studies showed moderate risk for bias due to missing data (D5) and some uncertainty regarding bias due to selection of participants (D2) and deviations from intended interventions (D4). Overall, approximately 75% of included observational studies demonstrated low risk of bias, with 20% showing moderate risk and only one study (8%) showing serious risk of bias.

(A)


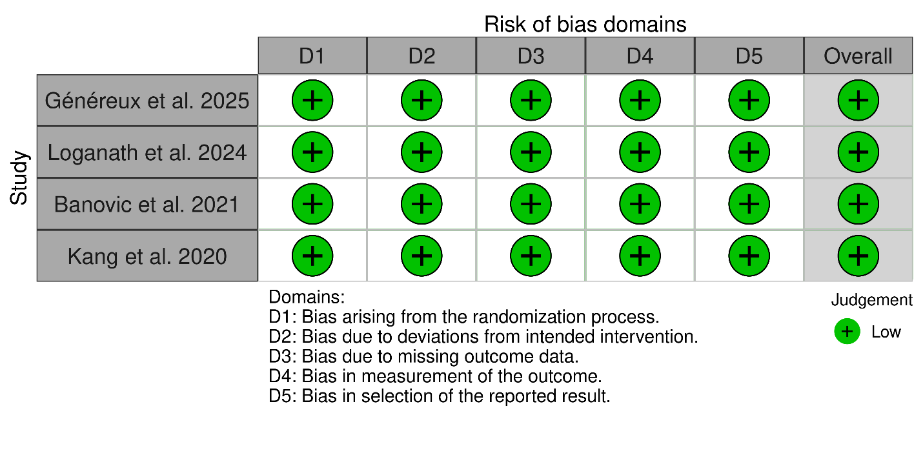


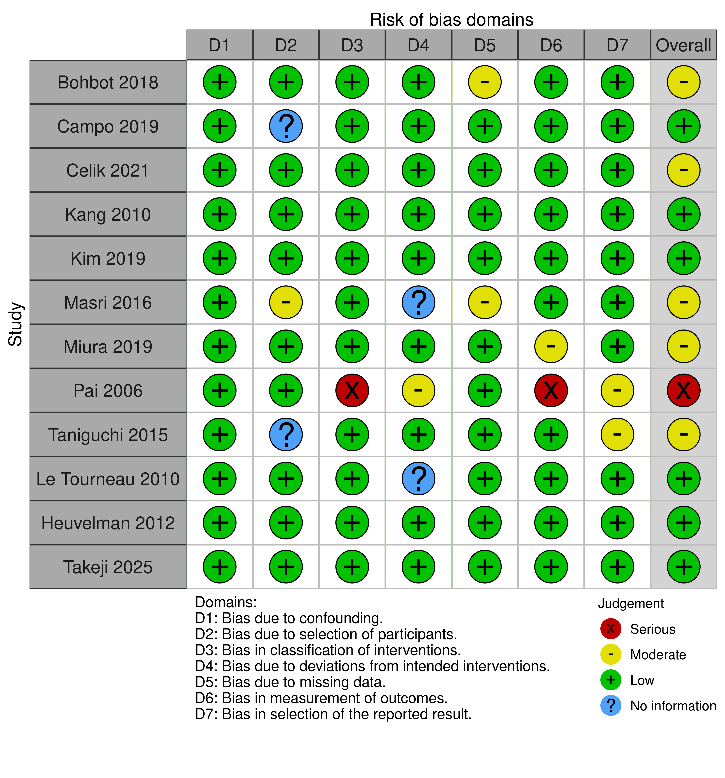
(B)


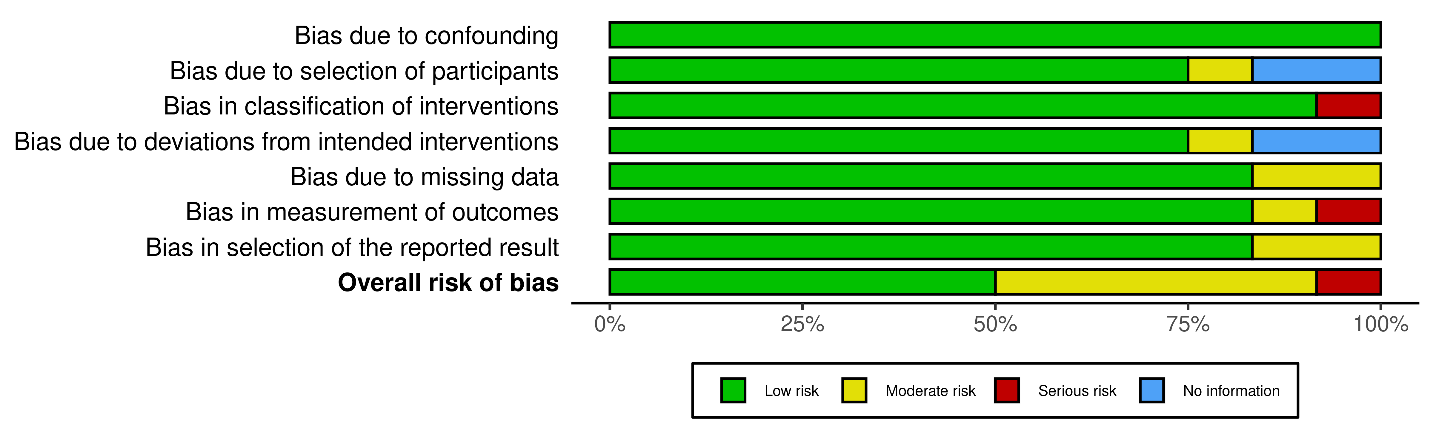
(C)

**Figure S3.** Forest plot of leave-one-out sensitivity analysis regarding (A) All-cause mortality (B) Cardiovascular death (C) HHF (D) sudden cardiac death (E) MI (F) Stroke


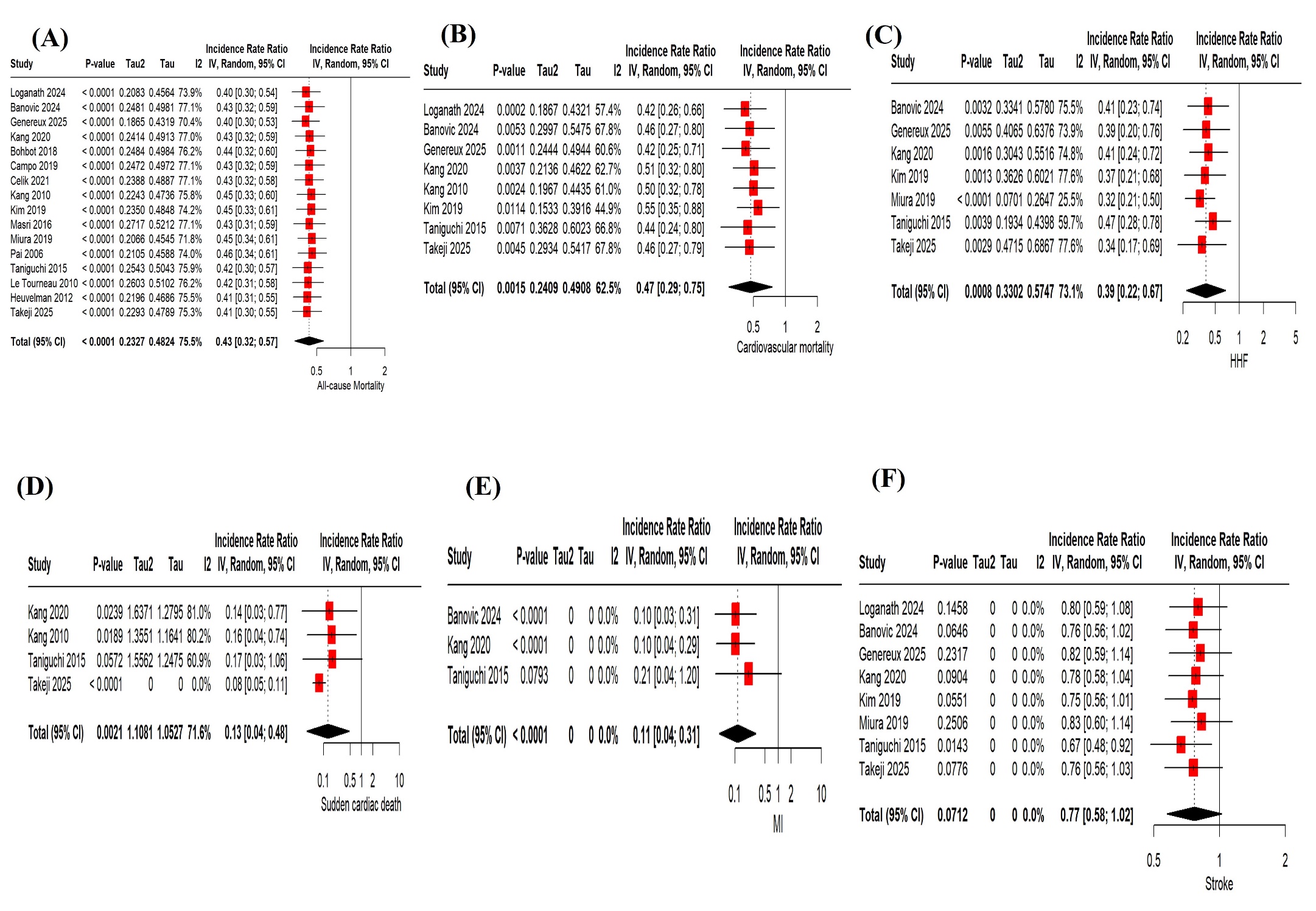


**Figure S4.** Testing PH Assumption using Schoenfeld Residuals.


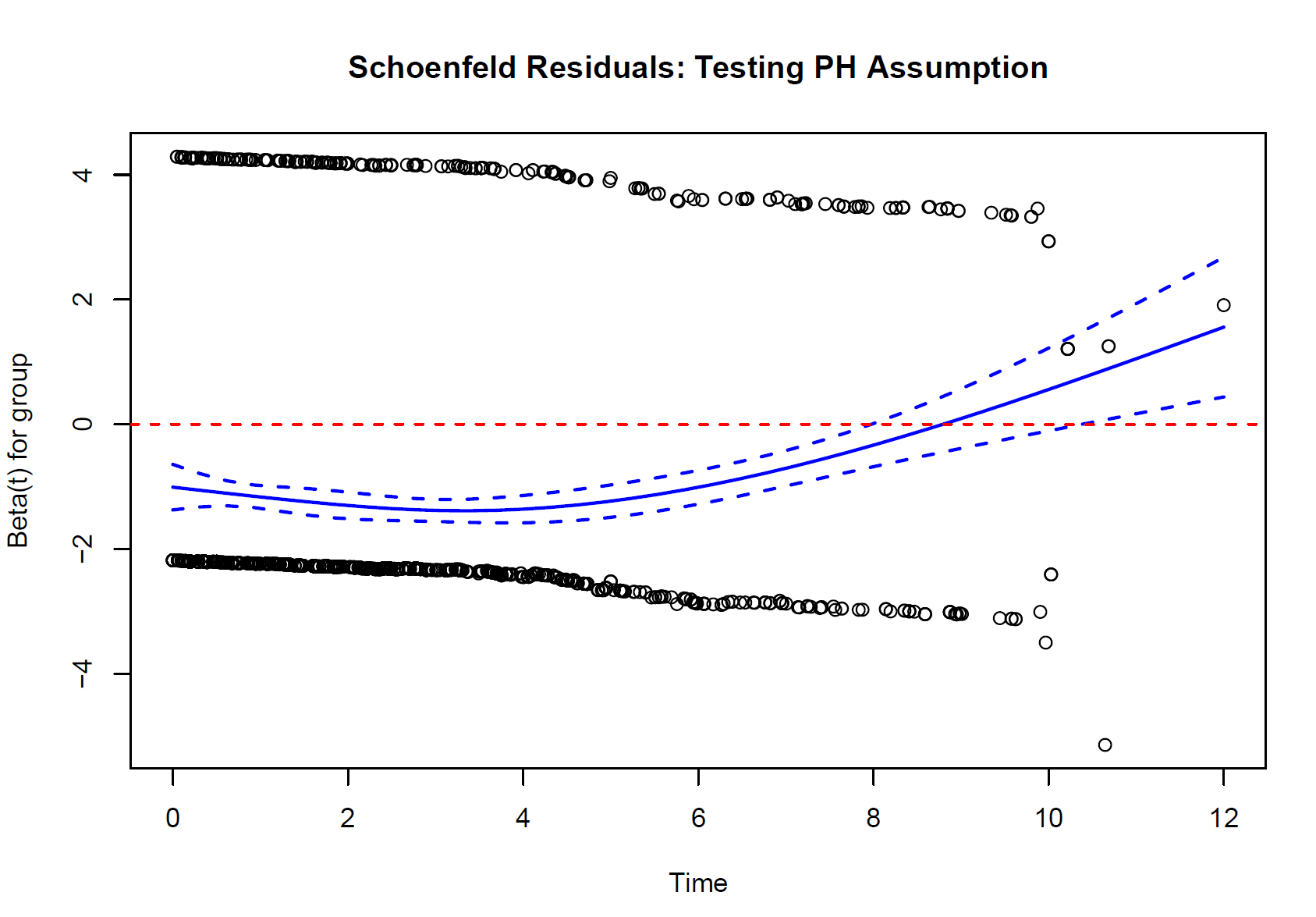


**Figure S5.** Time−Dependent Hazard Ratio analysis of Kaplan-Maier curve.


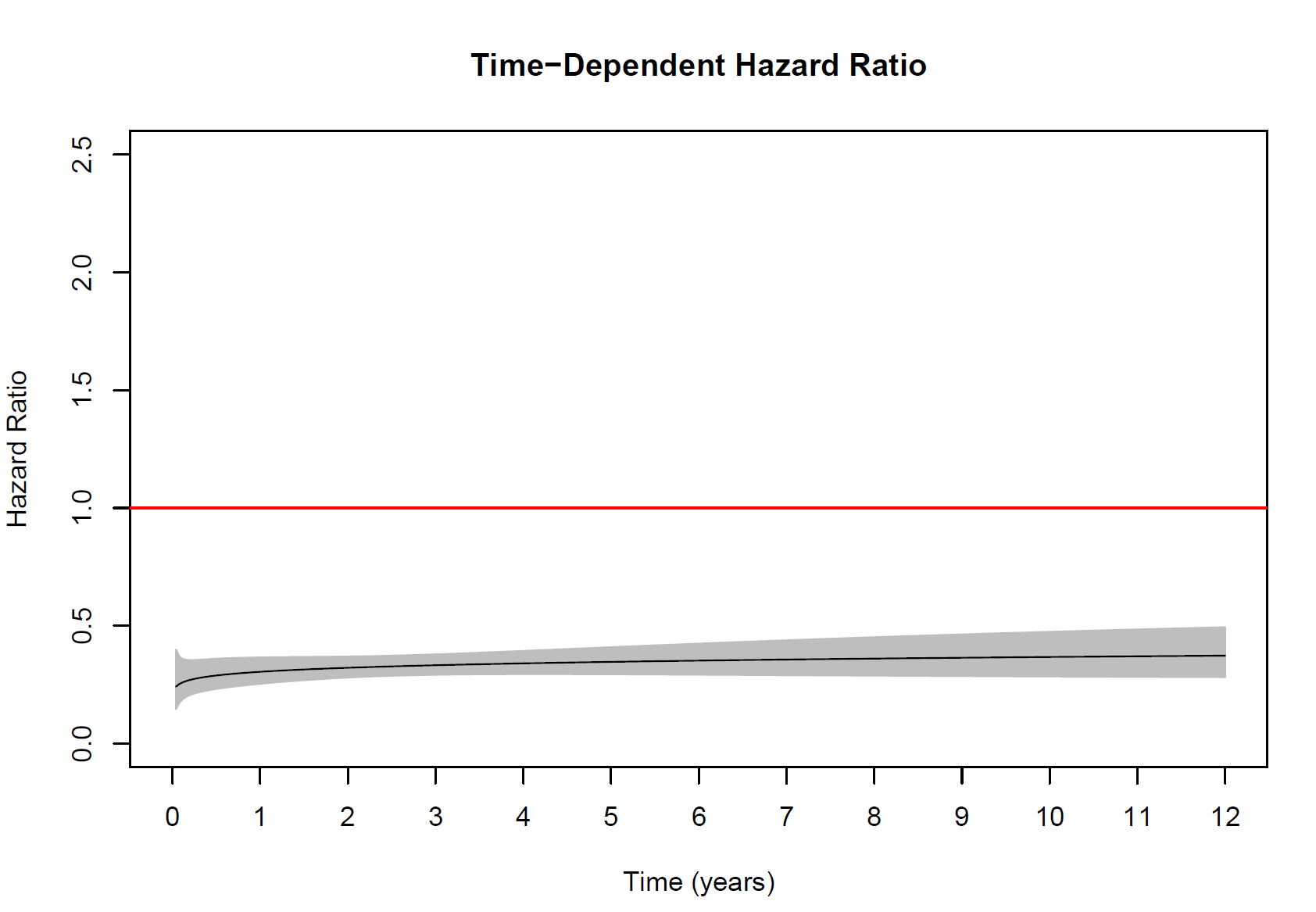


**Figure S6.** Reconstructed KM curve from randomized controlled trial data only.


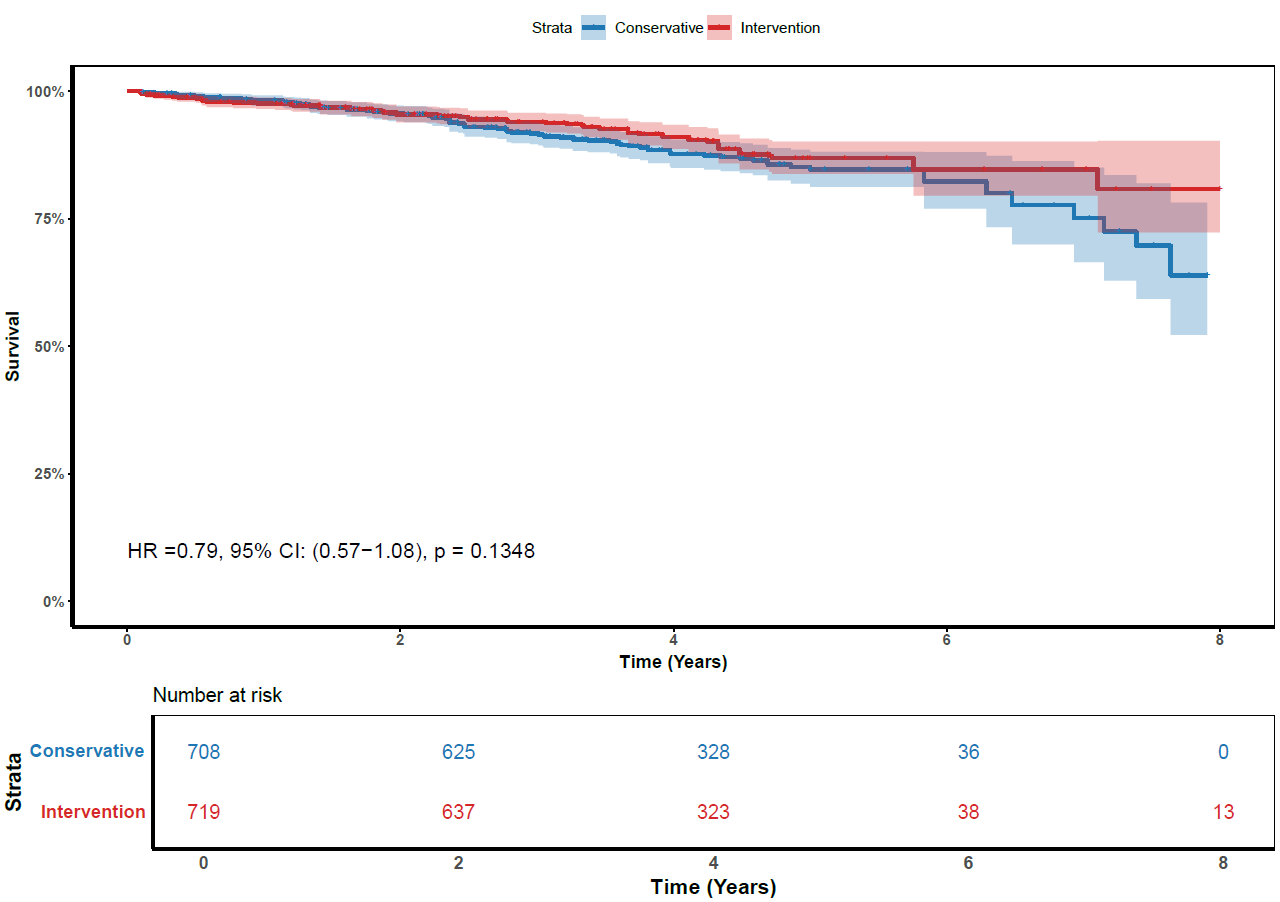

Supplement: ivaf308_Supplementary_Data [file ivaf308_supplementary_data.zip › Supplementary_revised.docx]
